# Trade of economically and physically scarce virtual water in the global food network

**DOI:** 10.1038/s41598-021-01514-w

**Published:** 2021-11-23

**Authors:** Elena Vallino, Luca Ridolfi, Francesco Laio

**Affiliations:** Department of Environment, Land and Infrastructure Engineering, Politecnico di Torino, Corso Duca degli Abruzzi 24, 10129 Turin, Italy

**Keywords:** Environmental sciences, Environmental social sciences, Hydrology

## Abstract

The virtual water (VW) trade associated to food is composed by the quantity of water utilized for the production of the crops exchanged on the global market. In assessing a country’s water abundance or scarcity when entering the international VW trade, scholars consider only physical water availability, neglecting economic water scarcity, which indicates situations in which socio-economic obstacles impede the productive use of water. We weight the global VW trade associated to primary crops with a newly proposed composite water scarcity index (CWSI) that combines physical and economic water scarcity. 39% of VW volumes is exported from countries with a higher CWSI than the one of the destination country. Such unfair routes occur both from low- to high-income countries and among low- and middle-income countries themselves. High-income countries have a predominant role in import of CWSI-weighted VW, while low- and middle-income countries dominate among the largest CWSI-weighted VW exporters. For many of them economic water scarcity dominates over physical scarcity. The application of the CWSI elicits also a status change from net exporter to net importer for some wealthy countries and *viceversa* for some low- and middle-income countries. The application of CWSI allows one to quantify to what extent VW exchanges flow along environmentally and economically unfair routes, and it can inform the design of compensation policies.

## Introduction

Virtual water trade is constituted by the volume of water that is consumed for the production of goods that are subsequently traded on the international market^1–4^. The trade of agricultural products accounts for about 90% of the total virtual water (VW) displaced for human consumption^5^. The VW associated to traded food at the global level is approximately 25% of the total amount of water utilized for agriculture^5^, and it has doubled from 1986 to 2007^6^. Moreover, from the 1990s to 2015 the quantity of food exchanged on international markets has increased almost three times faster than food production^7^.

Along with increased globalization and higher importance of long value-chains in the food domain, the economic and environmental system tends more and more to a detachment between places of production and consumption^4,8^ and to an increased countries’ vulnerability^5,9–12^. Through the outflow of virtual water embedded in food exports, countries renounce to precious domestic water resources, while through the inflow of VW included in food imports, countries benefit of water belonging to other areas of the world^2,4,12^.

Beside the reconstruction and the analysis of the structure of the VW network worldwide^6,13^, scholars have analyzed the global VW dynamics mainly according to two viewpoints. In the first one, authors address the question on whether VW trade compensates or not for lack of available resources (including water and land) for food production in given countries or areas^14–16^. Many scholars find evidence that VW trade alleviates pressure on several water stressed regions, which are allowed to import and consume food that has been produced in other world regions where water is more abundant^1,14,17–20^. On the other hand, other authors identify some paradoxes since some water-scarce countries or areas actually are net VW exporters, and vice versa^21–25^. A second group of scholars focuses on whether VW fluxes associated to agricultural products are ‘sustainable’ from an environmental point of view, providing different estimates of water stress due to VW trade^18,26–33^. However, only physical water availability is typically considered in both viewpoints when the water endowment of a country is assessed, i.e. its ‘starting line’ in the scene of international virtual water trade^17^.

If sufficient physical availability of renewable water resources is the crucial pillar of productive water use^34–39^, the second pillar is represented by an infrastructural, economic and institutional environment that allows water access and use^40–42^. The lack of possibilities for water utilization due to social, economic and institutional factors is labeled by many scholars as economic water scarcity^43–45^. For example, in countries such as Congo DR and Cambodia water stress is low according to hydrological parameters, but indicators on water use efficiency or agricultural performance depict a critical situation, leading to the intuition that the utilization of available water is far from effective^46–48^. In some Central Asian countries, such as Afghanistan or Kazakhstan, disinvestment in irrigation infrastructures led to waste of water resources and to economic losses in agriculture, although the physical pressure on hydrological resources is comparatively low^49^. Therefore, underinvestment in water institutions and infrastructures and weak water governance processes usually seriously prevent a proficient water use in different sectors and in particular in agriculture. According to some estimates, about 1.2 billion people live in zones of physical water scarcity, while another 1.6 billion people is located in areas of economic water scarcity. Moreover, low-income households tend to suffer more from consequences of economic water scarcity^49^, p. 48. The notion of economic water scarcity has been studied by both international organizations^50,51^ and academic scholars^44,45,52^, in order to reflect on common definitions and to design appropriate indicators for its measurement and quantification^31,52,53^. The aim has been to consider organizational issues, political accountability, infrastructure and institutions for water access, beside physical water availability. This investigation overlapped with other streams of research that addressed the same issue from slightly different angles. Scholars working on the water poverty concept aimed at combining information on estimates of water presence in a country or area with variables that reflects socio-economic poverty^43,52,54^. Other authors worked extensively on multidimensional water security, combining indexes on water availability, accessibility, safety and management^41,42,52,55^. The discipline of socio-hydrology addresses the interaction between the dynamics of hydrological resources and of social human processes in order to study water systems and to estimate associated risks^40^. Due to the inherent complexity of the phenomenon of economic water scarcity, its measurement is particularly challenging and only recently attempts have been made to quantify this dimension and to estimate its impact on agricultural performance following data-driven approaches^53,56^.

Within this picture, in the present paper, we reassess the VW volumes associated to the international trade of primary crops under the lens of both physical and economical water scarcity of the country of origin. We argue that it is appropriate to consider economic water scarcity (EWS) in the context of trade because EWS, exactly as physical water scarcity, is a feature that characterizes the country in which the traded crop is cultivated, e.g. one of the endowments of the factors of production of a country. Through the virtual water inflow from areas with high water scarcity, importing countries compete with the local population for the use of the hydrological resources that are physically and economically available in the producing country.

As we will explain more in detail in Sect. 2 and in the Data and Method section, we consider the volumes of green and blue water that are used in each country for the cultivation of the primary crops that are traded internationally. We consider 2016 since it is the last year for the availability of virtual water data from the CWASI database^46^. We weight those volumes with an index that reflects, for the respective country, the lack of available water (physical water scarcity) and the lack of management and governance practices that improve economic and infrastructural access to hydrological resources (economic water scarcity). This weighting methodology has been utilized by Lenzen et al.^27^ who, however, apply only the physical water scarcity weight, while we apply a composite water scarcity weight that includes also economic water scarcity. As^27^ underline, we utilize water scarcity information only as an input into a weighting procedure, in order to convey the message that the virtual water associated to traded food has been used in countries facing different degrees of water scarcity, due to either physical or socio-economic causes, or both.

For the first time, to the best of our knowledge, a novel viewpoint for the consideration of water scarcity is adopted. We provide a quantitative measure of the VW flows that are considered scarce under this new perspective and an assessment of the changes that this new metric produces in the role of the countries in the international VW trade, also in relation to their economic wealth.

## Results

### A composite water scarcity index

**Figure 1:**
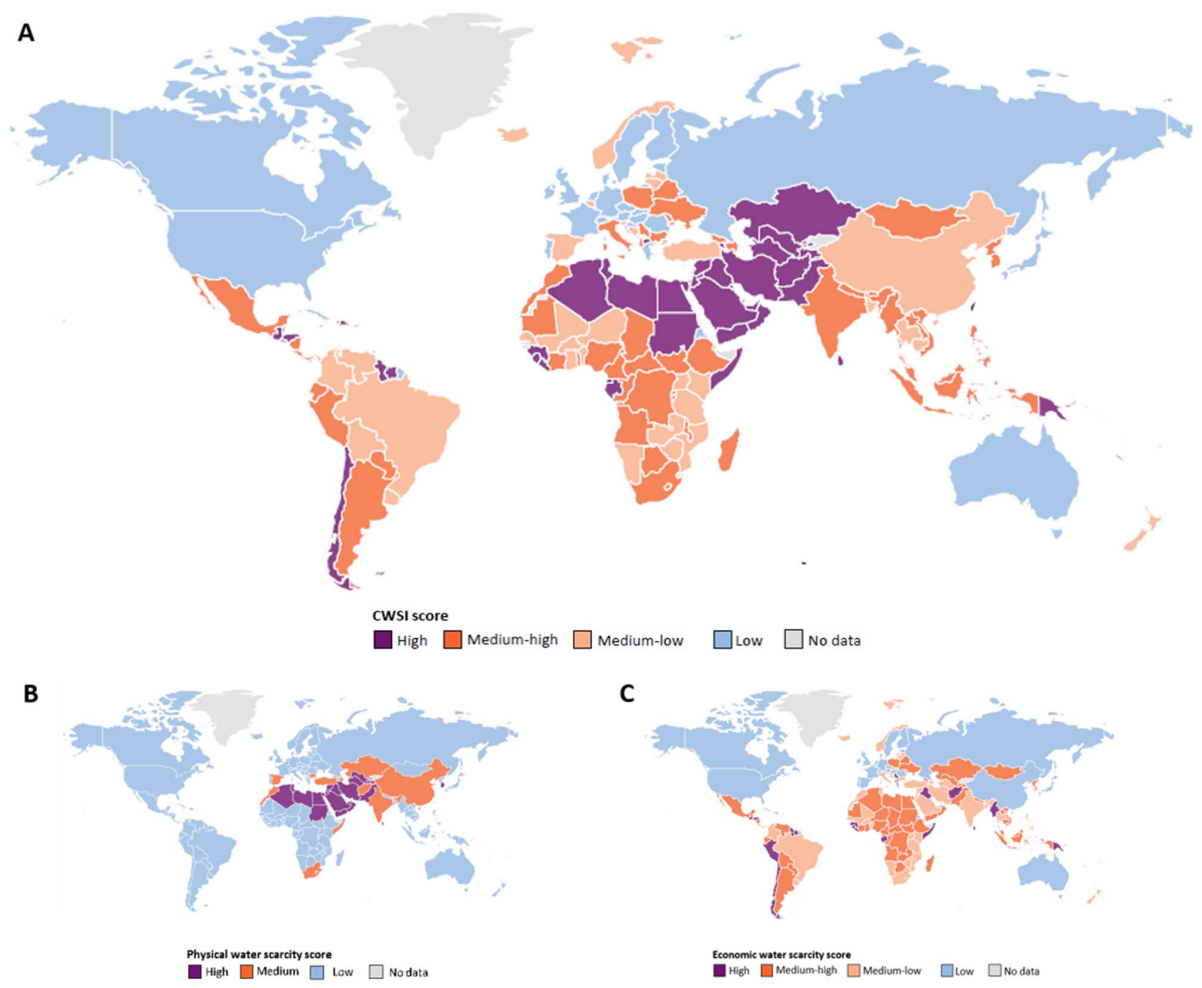
(**A**) Composite water scarcity index (CWSI) in 2017. Low, medium–low, medium–high and high scores correspond to intervals (0, 0.35), (0.35, 0.55), (0.55, 0.75), and (0.75, 1), respectively. (**B**) Physical water scarcity index in 2017. Low, medium, and high scores correspond to intervals (0, 0.2), (0.2, 0.4), and (0.4, 1), respectively. Thresholds are retrieved from the scientific literature[52,60–62]. (**C**) Economic water scarcity index in 2017. Low, medium–low, medium–high and high scores correspond to intervals (0, 0.30), (0.30, 0.50), (0.50, 0.70), and (0.70, 1), respectively. Thresholds are retrieved from the official documents related to the IWRM index^57^. The map was generated with D3.js (authors elaboration; https://d3js.org Version 4.13.0).

**Figure 2:**
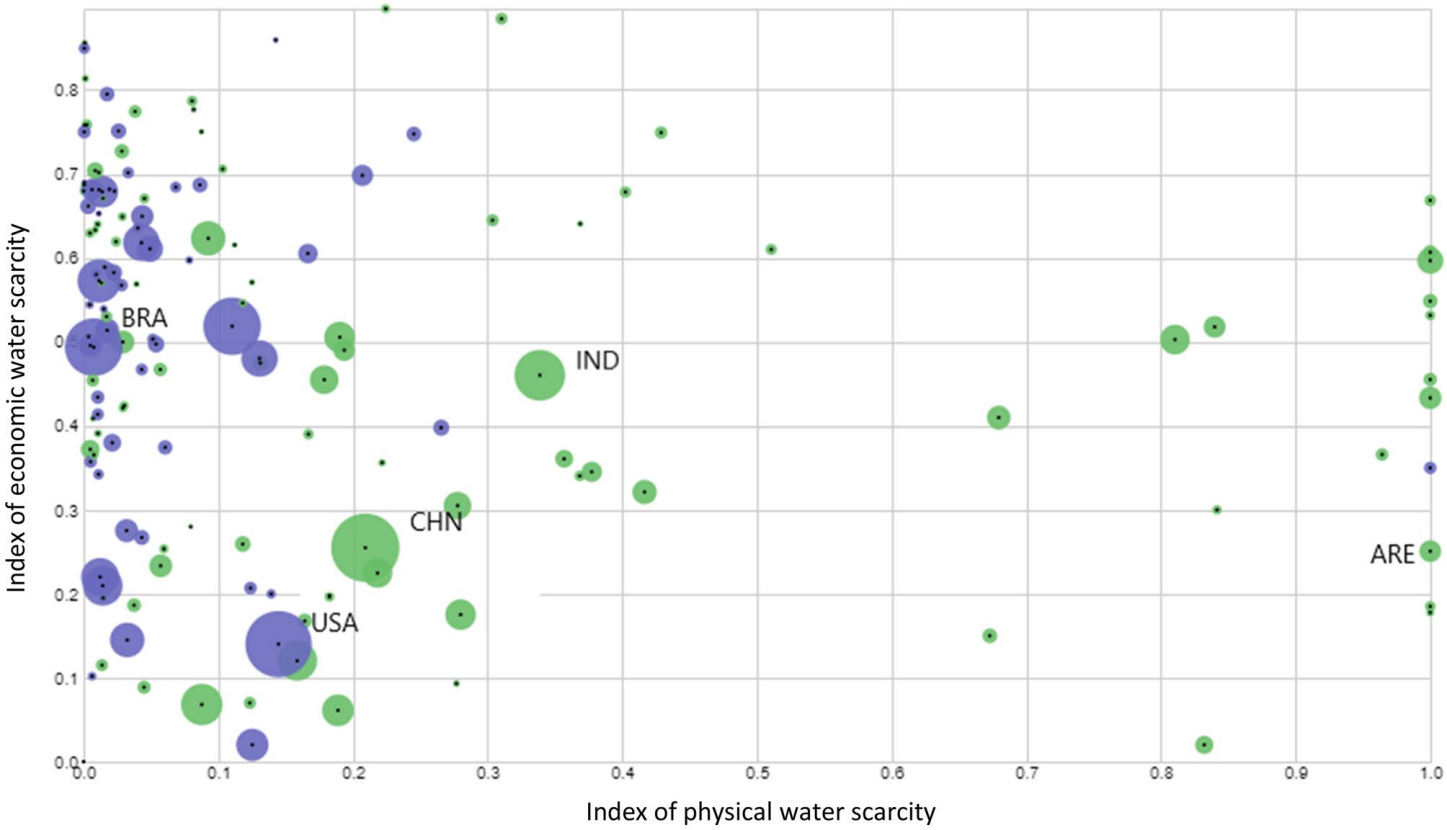
Physical water scarcity (hydrological pressure) and economic water scarcity (1 − IWRM) for 149 countries (2017). The size of the point is proportional to the total volumes of VW involved in both export and import for that country (m3). Green points indicate net importer countries (having VW import associated to food higher than export), while violet points indicate net exporter countries (having VW export higher than import). The ISO codes correspond to the following countries. *BRA* Brazil. *USA* United States of America. *CHN* China. IND: India. *ARE* United Arab Emirates.

We build a composite index of physical and economic water scarcity. In order to account for the physical water scarcity (PWS), we utilize the indicator of hydrological pressure that quantifies the percentage of freshwater withdrawal over the total renewable water resources in a country (see Supplementary Table S4). This indicator is provided by ACQUASTAT^47^, it has already been utilized by Lenzen et al.^27^ and discussed in different reviews, including^41,52^. It is therefore a used and well acknowledged indicator^52^, and it is of immediate understanding. For this index, zero is associated to absence of pressure, while 1 denotes maximum pressure. For the quantification of economic water scarcity we use the SDG indicator 6.5.1 on Integrated Water Resource Management—IWRM, (see Supplementary Table S4)^57^. The indicator reflects different dimensions of water governance and management, providing a framework to assess whether, in a given country, water resources are managed in an equitable, efficient, and sustainable mode. It is based on a set of questions organized in four pillars posed to country representatives: enabling environment, institutions and participation, management instruments and financing^57^. The adequacy of this indicator for the measurement of economic water scarcity has been assessed in^53,58–60^ Vallino et al.^53^, and UN Environment^57^ estimated also that the indicator brings along new information with respect to the most common measures of socio-economic development of a country, such as the Gross Domestic Product per capita and the Human Development Index (see also^52^). The IWRM is close to zero when low levels of water management are present (i.e., economic water scarcity is maximum) and to 1 when best water management is attained (i.e., economic water scarcity is absent). Both indicators cover 90% of the countries of the world and approximately 99% of the world population (see Supplementary Table S4). Different scholars provided valuable reflections on the strengths and challenges of both indicators^27,41,52,53,58–60^, as we discuss in the Data and Method section. The composite water scarcity index (CWSI) that we propose, ranging 0–1, is constructed as

1 $$ CWSI = 1 - IWRM\left( {1 - PWS} \right) $$

where *PWS* is the physical water scarcity measured through the above-mentioned index of hydrological pressure. The CWSI is equal to 1 when scarcity is at the maximum level, and this occurs when either the physical scarcity or the economic scarcity attain the maximum stage (i.e., IWRM = 0 or PWS = 1). Conversely, it is closer to zero when both the economic scarcity and the physical scarcity are low (i.e., IWRM = 1 and PWS = 0) (see Supplementary Table S4). Figure 1A shows the distribution of the CWSI in the world in 2017, which is the last year when the IWRM is available^57^. The highest values, denoting highest composite water scarcity, are concentrated in the MENA region and in Central Asia, with hot-spots also in Central and South America. The lowest values are mainly located in high-income countries, with some exceptions, such as Italy and some countries in East and South-East Europe which present relatively critical levels of economic water scarcity despite belonging to the high-income group. Norway and Iceland are examples of water abundant countries but have a level of water management that is not best according to the IWRM indicator, while Spain suffers from water scarcity mainly from a physical point of view, having conversely a high level of water management. Many countries, such as Congo DR, Chile and Myanmar, have high water availability, but their low level of water management leads to a high value of the composite scarcity index, denoting a critical situation that would have been overlooked if the dimension of economic scarcity had not been included. Figure 1B, C show the physical water scarcity index and the economic water scarcity index, respectively, where the latter is computed as 1-IWRM. In Fig. 2 we observe that there is not a significant correlation between the indexes of physical and economic scarcity, as assessed, among others, by Damkjaer and Taylor^52^, therefore our CWSI index brings along and condense new significant information.

### Virtual water flows between countries with large difference in composite scarcity

We aim at assessing the VW trade associated to primary crops under the lens of our composite water scarcity index, by quantifying the VW volumes flowing between countries with different water scarcity situations. Therefore, firstly we sum the blue and green VW volumes associated to the trade of 123 primary crops exchanged by every country pair in 2016, which is the last year available in the VW trade matrix developed in the CWASI database^46^ (see Data and Methods and the Supplementary Material). In our matrix we include 151 countries and 22,200 VW fluxes encompassing a total of 1.27E + 12 VW cubic meters (See Supplementary Materials).

In order to quantify the difference in the composite water scarcity for the partners involved in VW trade associated to primary crops, we calculate the gap (*Gap*_*CWSI*_) between the CWSI of the exporter country (*e*) and that of the importer (*i*) for all bilateral VW trade flows. For every VW flow, the gap is calculated as

2 $$ Gap_{CWSI} = CWSI_{e} - CWSI_{i} $$

A positive gap denotes that the exporter has a more severe composite scarcity than the importer, and vice versa. We group the index gaps in 20 classes with a width of 0.1 points each. In our view, ‘unfairness’ occurs when VW is exported from a country that has a higher composite water scarcity than the importer, because in this case water is more scarce and precious for physical and/or socio-economic reasons in the origin country than in the destination country. By importing water from places with high economic water scarcity, importing countries are competing with local communities for benefitting from the water resources that actually are physically and economically available in the producing country. Conversely, a country with lower water scarcity is more in the position of exporting virtual water to a country facing more scarcity, and for this reason, facing more difficulty in producing crops domestically. In this case we label the VW exchange as ‘fair’.

**Figure 3:**
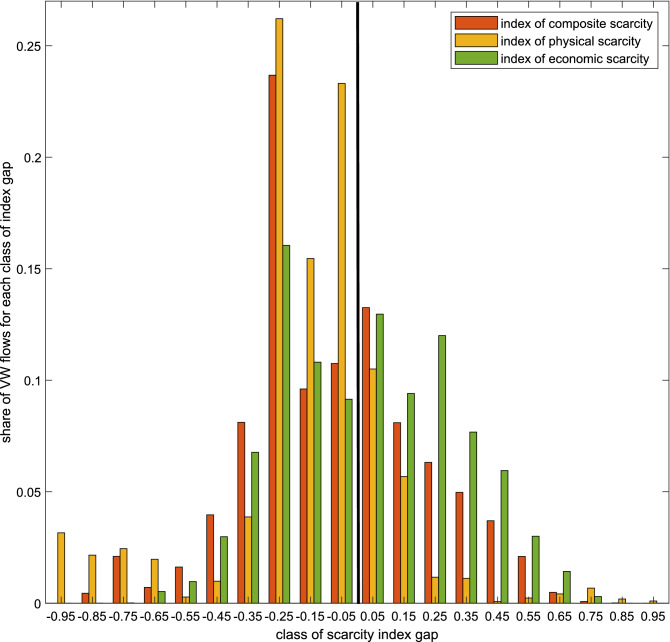
Share of VW fluxes for every class of scarcity index gap for all bilateral VW flows (2016). The gaps have been calculated for the composite scarcity index (CWSI, red bars), for the physical scarcity index (yellow bars), and for the economic scarcity index (green bars).

In Fig. 3 we represent the share of VW flows included in each of the 20 classes of the index gap, calculated over the total amount of VW trade. We find that 39% of the water volumes exchanged for the primary crops take place between countries with a positive gap. This means that this amount of virtual water is exported from countries with a higher composite water scarcity than the one of the destination countries, suggesting an ‘unfair exchange’, where the importing country benefits from the water of another area of the world where this resource is scarcer either in physical, or in economic terms, or both. Wecompare the status quo with an ideal artificial scenario in which all fluxes are fair, and in which fluxes logically follow the principle that virtual water flows from water-abundant (from both a physical and economic point of view) to water-scarce countries. In this perspective, our result of 39% of unfair fluxes over the total denotes a relatively high prevalence of unfairness.

In particular, the total volume exchanged between countries for which the difference between the respective scarcity indexes is positive and large (≥ 0.45, so strongly ‘unfair’), is equal to 6.4% of the world total (9.755E + 10 m3).

We repeat the procedure of Eq. 2 by substituting the CWSI with the economic water scarcity index (EWS, computed as 1-IWRM) and with the physical water scarcity index (PWS). As we observed in Fig. 2, the dynamics related to the two kinds of scarcity do not overlap. For VW flowing along ‘unfair’ routes (large and small positive gaps), economic water scarcity dominates over physical scarcity. In fact, 53% of VW volumes flow between countries with a positive gap in the EWS index, while only 20% flow between countries with a positive gap in the PWS index. On the contrary, physical scarcity dominates along ‘fair’ routes, with 80% of VW volumes flowing between countries with a negative gap in the PWS index and only 47% between countries with a negative EWS index gap. The majority of the physically-scarce VW is displaced between countries that are similar from that perspective (small gaps for physical water scarcity). Economically-scarce water instead is more distributed among both similar countries and countries with a larger gap from that point of view.

By adding the economic wealth dimension to the analysis (Supplementary Figure S1), we note that the higher number of large bilateral VW fluxes is concentrated in a space of positive CWSI index gap and negative Gross National Income per capita (GNIpc) gap (where the GNIpc gap is denoted by the difference between the exporter’s and the importer’s GNIpc)^64^. This space is located in quarter IV in Supplementary Figure S1, where both gaps represent a disadvantaged situation of the exporter country with respect to the importer country.

### Single fluxes of scarcity-weighted virtual water.

In order to study the VW trade from the point of view of the composite water scarcity, following Lenzen et al.^17^, we start by applying the CWSI weight, placed in the range 0–1, to every bilateral VW flux associated to primary crops in 2016, as expressed in the following equation:

3 $$ WF_{ij} = CWSI_{i} *F_{ij} $$

where $F_{ij}$ represents the VW flux traded from country *i* to country *j,* and $WF_{ij}$ is the weighted flux.

**Figure 4:**
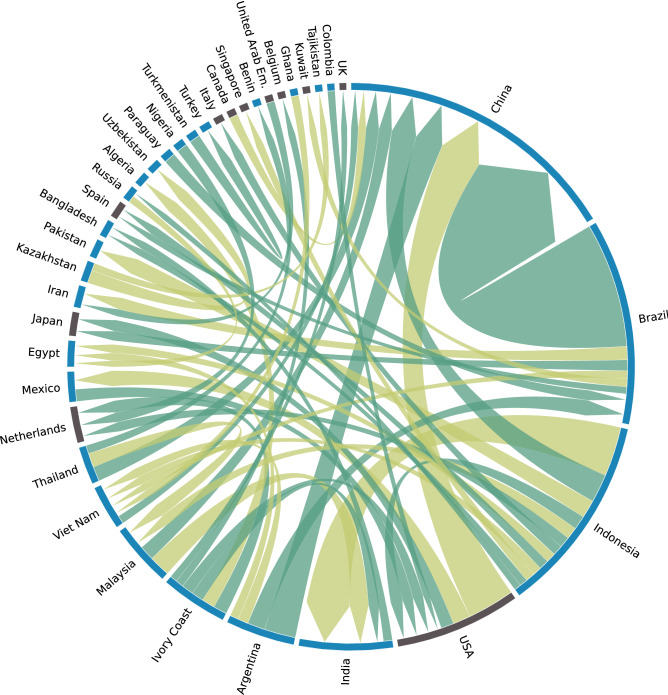
Largest 50 scarcity-weighted VW fluxes (CWSI-weighted m3). The flux flows in the direction of the arrow. Unfair fluxes (fluxes for which the exporter country has a higher composite scarcity index than the importer) are coloured in dark green; fair fluxes are in light green. High-income countries are in black; low-and middle-income countries are in blue.

In Fig. 4 the 50 largest single CWSI-weighted VW fluxes are represented, that together compose 36% of the world total CWSI-weighted VW traded. The three largest are those from Brazil to China (almost 6% of the world total), from Indonesia to India (2.3% of the total), and from USA to China (2.2% of the total). For many of the largest 50 scarce-water exchanges, VW fluxes are ‘unfair’, namely the CWSI gap between the involved countries is positive. This applies for example to the fluxes from Brazil to China, to Japan, and to Spain, from Indonesia to China, or from Ivory Coast to China, to India and to USA. Half of the ‘unfair’ fluxes of this group flow from low- or middle-income countries to high-income countries, and the other half flow among low- and middle-income countries, according to the income classification of The World Bank^64,65^ (see Data and Methods). The application of the CWSI weight to the VW fluxes generates important changes with respect to the unweighted case (See Supplementary Table S1). The second largest VW flux, from Indonesia to India, raises to the involvement of 2.3% of the world total scarce VW fluxes, while it represented only the 1.9% of the total volumetric VW fluxes. On the contrary, the flux from USA to China decreases its position, from representing the 4% of the world total VW trade in the volumetric case to the 2.2% in the CWSI-weighted case. Some fluxes, such as from Malaysia to China, and from Indonesia to Pakistan, enter among the top 10 largest fluxes if the CWSI weight is applied, replacing the fluxes from Netherlands to Germany and from Canada to China. Half of the 20 largest fluxes increase their position in the world ranking in the CWSI-weighted case. This means that many of the largest VW fluxes associated to primary crops in the world has origin in countries with high composite water scarcity.

### Largest players in scarcity-weighted VW trade

After having highlighted the largest single bilateral fluxes of CWSI-weighted virtual water, we focus on the features of the countries playing the major roles in the CWSI-weighted VW trade. We assess the balance between how much a country exports domestic scarce VW or it imports water that is scarce for others from a composite perspective. We also analyze the changes in the country rankings when the CWSI is applied to their virtual water fluxes.

For each country *i*, we measure the scarcity-weighted VW export (*WE*_*i*_) as

4 $$ WE_{i} = CWSI_{i} *\mathop \sum \limits_{j} F_{ij} $$

where $F_{ij}$ are the VW fluxes traded from country *i* to country *j*. Conversely, we obtain the scarcity-weighted VW import for country *i* (*WI*_*i*_) by weighting its imported VW volumes from each trading partner *j* with the CWSI of the partner *j*:

5 $$ WI_{i} = \mathop \sum \limits_{j} CWSI_{j} F_{ji} $$

We obtain the status (net importer or net exporter) of the country by simply calculating the difference between its export and its import, for both volumetric values and for CWSI-weighted values, and by normalizing the results for comparability purposes (see Data and methods). We repeat the whole procedure by substituting the CWSI with, respectively, the index of physical scarcity (PWS) and the index of economic scarcity (EWS, expressed as 1-IWRM) in Eqs. (4) and (5) (See Supplementary Material).

#### Scarcity-weighted net VW trade in absolute figures

Table 1 represents the share of each country’s net import and net export in absolute figures (left layer; *SN* and *SWN* in Eqs. 9 and 10 in Data and Methods) and in per capita terms (right layer; *sn* and *swn* in Eqs. 13 and 14 in Data and Methods). In general, we detect large gaps between volumetric VW values and scarcity-weighted VW values for the countries’ net status. In particular, comparing volumetric net VW export and the same metric weighted for the CWSI (Table 1, left layer) we note that Indonesia shifts from the third to the first position, while in the fourth and the fifth positions Argentina and Australia are replaced by Ivory Coast and Malaysia. Ukraine, Paraguay and Kazakhstan enter the top 10 group for the CWSI-weighted ranking, while they are absent from the top 10 of the volumetric ranking. Countries that increase their position in the CWSI-weighted ranking belong mainly to the low- and middle-income group and the shift is mainly driven by economic water scarcity (For figures on separate EWS and PWS weights see Supplementary Table S2).

**Table 1:** Top 10 countries for net VW export and import, in volumetric values and weighted for the composite water scarcity index.

		Volumetric values		Composite scarcity weight				Volumetric values		Composite scarcity weight	
Total (world share)	Net exp	Brazil	9.27	Indonesia	9.71	Per capita (world share)	Net exp	Uruguay	5.31	Paraguay	6.81
		USA	8.24	Brazil	9.46			Paraguay	5.09	Ivory Coast	3.88
		Indonesia	7.39	Argentina	5.71			Australia	3.82	Uruguay	3.87
		Argentina	4.26	Ivory Coast	4.03			Ivory Coast	2.83	Argentina	3.11
		Australia	3.73	Malaysia	2.78			Canada	2.55	Moldova	2.89
		Canada	3.71	Thailand	2.66			Lithuania	2.44	Turkmenistan	2.84
		Russian F	2.77	Ukraine	2.41			Argentina	2.42	Kazakhstan	2.37
		Ivory Coast	2.73	USA	2.04			Moldova	2.08	Lithuania	2.14
		Thailand	2.15	Paraguay	2.01			Malaysia	1.73	Malaysia	2.08
		Malaysia	2.15	Kazakhstan	1.84			Bulgaria	1.68	Bulgaria	1.89
	Net imp	China	− 16.01	China	− 15.1		Net imp	Netherlands	− 3.81	Netherlands	− 5.83
		Japan	− 3.38	Netherlands	− 4.33			Belgium	− 3.12	Belgium	− 2.69
		Germany	− 3.08	Germany	− 3.12			Singapore	− 2.71	Singapore	− 2.51
		Netherlands	− 2.62	Japan	− 2.74			Un. Arab Em	− 1.66	Oman	− 1.45
		Italy	− 2.18	Turkey	− 2.26			Israel	− 1.34	Benin	− 1.11
		Turkey	− 2.07	Italy	− 2.09			Oman	− 1.31	Mauritius	− 1.09
		Spain	− 1.94	Spain	− 2.01			Saudi Arabia	− 1.11	Qatar	− 1.01
		Egypt	− 1.92	Korea, Rep	− 1.68			Kuwait	− 1.11	Saudi Arabia	− 1.01
		Korea, Rep	− 1.82	Iran	− 1.62			Mauritius	− 1.05	Spain	− 0.99
		Viet Nam	− 1.68	Egypt	− 1.45			Spain	− 1.03	Portugal	− 0.96

The left layer is referred to absolute figures (*SN* and *SWN*, in percentage of the world total). The right layer is referred to per capita figures (*sn* and *swn*, in percentage of the world total).

Regarding the net VW import (Table 1, left layer), the Netherlands increases its ranking if the CWSI weight is applied. Although Egypt decreases its position if composite water scarcity is taken into account, the decrease would be much stronger if economic scarcity were not included in the CWSI (Supplementary Table S2). Nevertheless, the largest players in this group do not drastically change their position between the volumetric and the CWSI-weighted rankings, and this implies that overall the largest net VW importers mainly import scarce water. For Japan, China, Germany and the Netherlands the large import of composite scarce water is driven by economic water scarcity.

#### Scarcity-weighted net VW trade per capita

**Figure 5:**
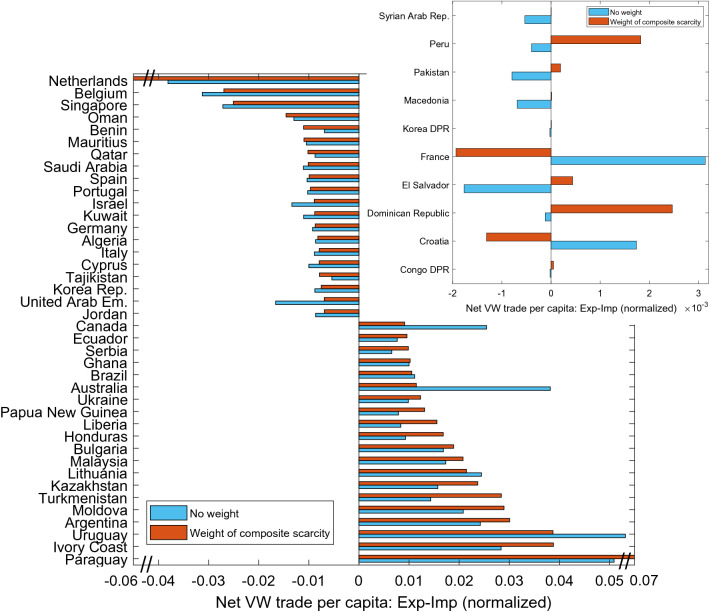
Largest 20 net CWSI-weighted VW per capita exporters (positive values for *sn* and *swn*) and largest 20 net CWSI-weighted VW per capita importers (negative values for *sn* and *swn*), expressed as world share. Net trade is calculated as export minus import. Inset: countries changing status from net VW importer to net VW exporter per capita, and vice versa, if the weight of composite water scarcity is applied (CWSI).

We now consider the same figures in per capita terms (Table 1-right layer and Fig. 5), where the share of a country’s net import or net export is represented by the variable *sn*_*i*_ in the volumetric case and *swn*_*i*_ in the CWSI-weighted case (see Eqs. 13–14 of the Data and Methods section). We aim to eliminate the effect of having large and highly populated countries in the top positions for both volumetric and scarcity-weighted VW trade.

For the top net CWSI-weighted VW exporters per capita, 15 countries out of 20 belong to income classes from low- to upper-middle (e.g. Ivory Coast and Malaysia; Fig. 5). However, among large scarce VW per capita net exporters, we identify both water rich and arid countries from a hydrological perspective. This suggests that exporting water which is domestically precious, also in net terms with respect to imports, is a highly diffuse phenomenon in poor or middle-income countries, independently of the country level of water availability, management and access. Many countries that increase their position in the CWSI-weighted ranking with respect to the volumetric ranking have a low or a middle income, and the shift is driven by EWS (Table 1 and Supplementary Table S2). This means that they export more composite scarce water that they import, and that the scarcity is dominated by the economic dimension. Conversely, for the CWSI-weighted case many rich countries in the top 10 positions for the volumetric case, either decrease their position or drop from this group, such as Canada and Australia (Table 1, right layer).

While the composition of the group of the largest net CWSI-weighted VW importers in absolute terms is more differentiated with respect to economic wealth (Table 1, left layer), we notice that 85% of the main CWSI-weighted net VW importers per capita are high-income countries, with the Netherlands, Belgium, and Singapore at the top (Fig. 5 and Table 1-right layer). Using per capita values we observe more changes in positions with respect to the absolute terms between volumetric and CWSI-weighted rankings, denoting a higher amount of information elicited through the application of the CWSI. Nevertheless, also in this case we can affirm the most of the VW inflowing to the top net importers is composed by composite scarce water and this applies mostly to high-income countries, as it is observable in the similar length of the red and blue bars in Fig. 5 (negative values). Moreover, if values weighted for EWS and PWS separately are considered (see Supplementary Figure S2), 14 high-income countries are placed among the top 20 net scarce VW importers for the net import of economically-scarce VW (e. g. Belgium and Spain). Some rich countries, such as the Netherlands and Saudi Arabia, populate the top 10 ranking for both net import of physically-scarce and economically-scarce VW.

#### Changes between country positions in the volumetric and in the CWSI-weighted case

Within the group of 20 largest net exporters per capita, 15 experience a reinforcement of their position if the CWSI weight is applied to VW fluxes, with Turkmenistan, Honduras and Liberia having the largest gap between volumetric and scarcity-weighted values. This reinforcement concerns 7 out of the 20 largest net importers per capita, with Netherlands having the largest gap (Fig. 5).

Some countries shift their net VW trade position if the CWSI weight is applied (Fig. 5, inset). France and Croatia are net VW exporters regarding volumetric VW values per capita, but they appear as net importers for scarcity-weighted VW. This shift is driven by the economic water scarcity dimension, because if we apply the physical scarcity weight alone, the net exporter status is even reinforced. The opposite holds for some low- and middle-income countries, among which Congo DR and Pakistan, that shift from being net importers for volumetric VW to being net exporters for scarcity-weighted VW.

**Figure 6:**
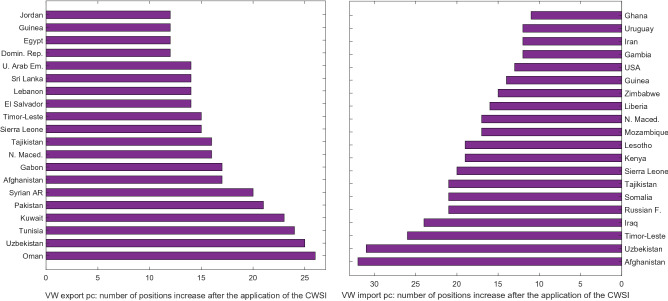
Left panel: 20 countries with the highest increase in position for VW export per capita if the CWSI is applied. Right panel: 20 countries with the highest increase in position for VW import per capita if the CWSI is applied. Rankings are on 149 countries.

Considering the whole world, in Fig. 6-left panel we observe the 20 countries for which the application of the CWSI generates the highest increase in position with respect to their ranking in volumetric VW export. In Fig. 6-right panel we consider the same metric referred to import. Most of the countries of both groups have an income from low to middle. Therefore, the consideration of composite water scarcity produces changes in perception on VW trade dynamics in several economically disadvantaged areas of the world, even if these countries are not included among the largest importers or exporters. Often EWS drives such large gaps, such as for Guinea and Sri Lanka for export per capita, or USA and Russia for import per capita. With the aim of quantifying the information brought by the economic scarcity weight on VW trade in the whole world (Supplementary Tables S3A and S3B), we notice that 65% of the net VW exporters per capita in the world have a larger net export for the economic-scarcity-weighted VW than for the volumetric VW. All these countries belong to the low- and middle-income group, with Paraguay and Ivory Coast having the largest gap (Supplementary Table S3A). 17% of the net VW importers per capita in the world have a larger net import for economic-scarcity-weighted VW than for volumetric VW. They are both high- and low-income countries, with Netherlands and Singapore presenting the largest gaps (Supplementary Table S3B).

**Figure 7:**
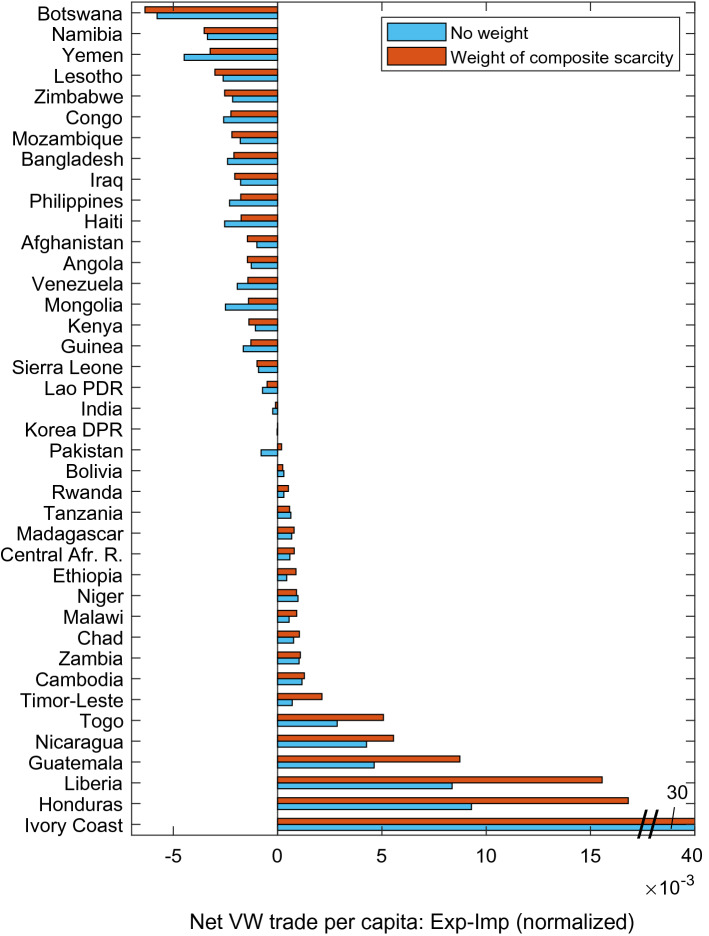
Net VW trade per capita status for 40 countries with the highest prevalence of undernourishment among the overall population, and with a composite water scarcity index higher than 0.5 (range: 0–1). Countries are ordered according to the share of CWSI-weighted VW trade per capita. Net trade is calculated as export minus import. Negative values refer to net importers and positive values refer to net exporters, expressed as world share.

If we focus on the 40 countries that suffer the most from food insecurity and from composite scarcity (see Data and Methods), we notice that overall these fragile countries export more scarce water and import less scarce water than it appears when we consider only volumetric VW values (Fig. 7). 15 out of the 18 net exporters of this category have a higher position if CWSI-weighted VW is considered (e.g. Ivory Coast and Guatemala), and 12 countries out of those 15 have an increase of more than 20% between shares in volumetric and scarce VW net export (e.g., Liberia and Timor-Este), with peaks of an increase higher than 100% in some cases such as Ethiopia and Honduras. This means that they overexploit for export scarce water resources that would be even more precious domestically, since they face serious food security issues. Moreover, they have fewer financial resources for food (and VW) purchase. Net VW exporters of this group mainly export economically-scarce VW (e. g. Tanzania and Nicaragua), even if some of them result as net importers of physically-scarce water (Supplementary Figure S3). Half of the net importers of this group import less CWSI-weighted VW than volumetric VW (e.g., Yemen or India).

### Focus on two emblematic cases: a net scarce VW importer and a net scarce VW exporter

**Figure 8:**
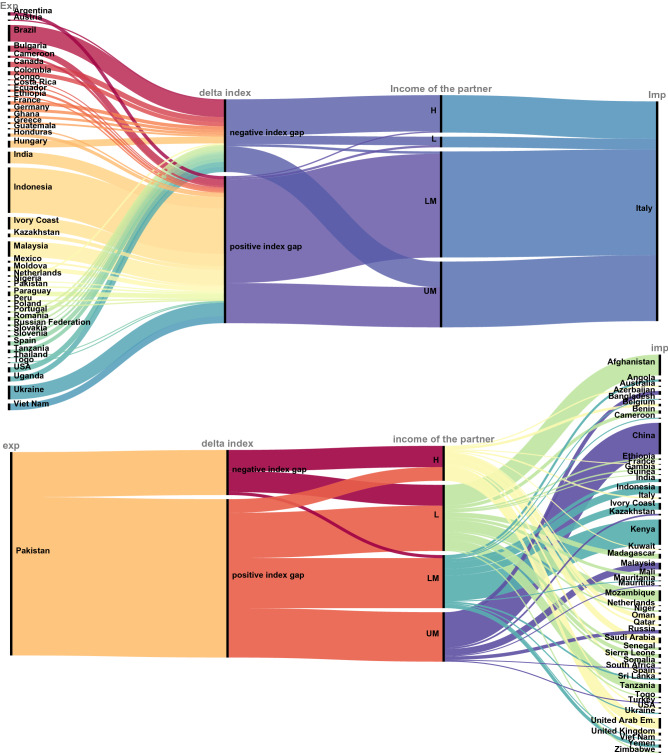
CWSI-weighted VW import for Italy (upper panel) and CWSI-weighted VW export for Pakistan (bottom panel). Left axis: Export. Right axis: Import. We included the fluxes representing 95% of the total traded volume (m3) by each of the two countries, both for export and for import. Positive index gap: exporter country has a higher composite water scarcity index than the importer (unfair exchange). Negative index gap: the exporter has a lower composite water scarcity index than the importer. *H* high income. *UM* upper middle income. *LM* lower middle income. *L* low income.

Italy is among the top 10 scarce VW importers in the world, in both absolute and net terms, and among the 20 largest net importers per capita of scarce VW (Fig. 8-upper panel), representing fairly well some patterns of high-income countries. Considering CWSI-weighted VW fluxes, exports are much lower and imports are much more concentrated from low- and middle-income countries than in the volumetric case. 60% of Italy’s 122 importing partners acquire a higher position if the CWSI weight is applied, and they are almost all from low- or middle-income countries, with Ivory Coast, Ukraine and Indonesia at the top. A positive gap occurs between the Italy’s CWSI and the indexes of more than half of the countries from which it imports scarce VW (Fig. 8-upper panel). This denotes a high prevalence of ‘unfair’ trajectories of VW flows where the composite water scarcity is higher in the exporting country than in the recipient country.

Pakistan is a paradigmatic case since it is both among the largest exporters and the largest importers of scarce VW. Interestingly it is among the countries with the largest difference between the volumetric figures and the CWSI-weighted figures for both import and export. However, it changes status from net VW importer per capita to net exporter per capita if the composite scarcity index is applied. Therefore, Pakistan can be utilized as representative of the cases for which a high amount of information is detected through the application of a composite water scarcity index, and as illustrative case for the group of low-and middle-income countries. The largest Pakistan’s partner for scarce VW exports is China (15% of the total) and exports are mainly directed to other low- and middle-income countries. For the large majority of the countries to which Pakistan exports, a positive gap exists between Pakistan’s CWSI and the one of the recipient country, denoting that Pakistan exports scarce VW to countries that comparatively suffer of lower levels of difficulty for water availability and access (Fig. 8-bottom panel). Overall, for scarcity-weighted VW trade, the role of Pakistan in import is reduced and its role in export is reinforced.

## Discussion and conclusion

The use of a composite water scarcity index (CWSI) that includes both physical and economic dimensions shows a picture that well describes the starting situation of a country and its degree of vulnerability with respect to water resources when it enters the arena of international virtual water trade.

Considering international bilateral trade fluxes of VW associated to primary crops we find that almost half of water volumes flow from countries that are worse-off with respect to recipient countries regarding composite water scarcity, with important overlapping with unbalances also in terms of economic wealth (e.g., from Mexico to the USA). This overlapping denotes the presence of ‘unfair exchanges’ and it suggests that the economic capability to compensate for the subtraction of precious water for export purposes may be lower for the countries at the origin of these flows. Apparently neither the income derived from the export of primary crops associated to these VW flows, nor the income derived from other sources constitute a compensation for the outflows of scarce water resources (as highlighted also by Dorninger et al.^8^). Moreover, more than half of world VW volumes flow from countries that are worse-off with respect to recipient countries regarding economic water scarcity.

The application of the CWSI generates large changes in positions of countries regarding their share in the global VW use for primary crop export, import and net trade, suggesting that the use of this index allows one to elicit a high amount of information.

High income countries have a predominant role in net import of scarce VW in per capita terms. For example, European countries such as the Netherlands, Italy, Germany, and Spain are among the major net scarce VW importers both in absolute and in per capita terms. For many of these countries the application of the CWSI reveals the largest gap between volumetric and weighted imported VW, and in some cases a status change from net exporter to net importer. Therefore, high-income countries do not only play a major role in importing water originated in other countries, but also in importing VW that is particularly precious for the countries it belongs to, due to the high vulnerability of the latter in terms of water availability and governance and of economic wealth. Most of the high-income countries in the top net importer group import mainly economically-scarce water.

Low- and middle-income countries dominate among the largest scarce VW exporters per capita. Countries of this category, such as Niger and Congo DR, also present the largest gaps between volumetric and CWSI-weighted VW export, with unbalance toward the latter. The application of the CWSI highlights a change of status for many of them from being net importers to being net exporters (e.g. Peru, Pakistan, El Salvador and Dominican Republic). There emerges a clear tendency to utilize precious local water resources for primary crop export purposes. An overall tendency of export of economically-scarce water is observable, but at the same time it is prevalent an import of physically-scarce water resources from trade partners with a similar difficulty. However, by collapsing both information it emerges that considering the CWSI weight the role of VW import for these countries is reduced and the role of export is reinforced. These countries are embedded in a dense network of ‘unfair’ VW fluxes, with the exporter having a higher CWSI than the importer. A smaller group of these countries, composed by fragile nations in terms of food security and water endowment, export more VW and import less VW if the CWSI weight is applied to VW trade fluxes. Therefore, the harm that they do to themselves by exporting VW embedded in primary crops is higher than it appears when only unweighted figures of VW trade are considered.

Despite the largest players for scarce VW import (in total and per capita terms) are dominated by high-income countries, the application of the CWSI at a global scale reveals a more complex scenario, in which the group of 20 countries having the largest difference between unweighted and weighted VW import, with unbalance toward the latter, is composed by low- and middle-income countries (with exception of the USA) with different levels of physical water scarcity. Therefore, the utilization of the CWSI highlights exploitation dynamics also among countries with similar levels of difficulties in the economic and water domains. This applies in particular to the fragile countries group, for example to bi-directional flows between Pakistan and Afghanistan, or between Congo DR and its neighbor countries.

In summary, high income countries consume large volumes of water that for lower income countries is scarce from both physical and economic perspectives. Moreover, a large share of the primary crops that embed the imported VW in high income countries is devoted to crop processing or re-export^66^. The considerable economic value added created by these activities^8^ is channeled, among other uses, for purchasing further quantities of primary crops from the same countries, reinforcing in this way structural patterns of unequal use of scarce water at a global level. Therefore, the ‘unfairness’ associated to many VW exchanges appears as systematic and it is not simply a side-effect of the dense network of food trade of the globalized world. Sustainability challenges in the domain of water resources represent what Dorninger et al. (2021) call “predictable consequences of ecologically unequal exchange”^8^. Nevertheless, phenomena of mutual exploitation of scarce water resources among countries with similar levels of difficulties in the water and economic domain have a remarkable weight as well, complexifying the interpretation of the ‘unfair’ water trajectories. VW fluxes of this country group respond to the need of assuring food security that may be in danger because of high water composite scarcity, among other causes, and they may be therefore unavoidable. The lens provided by a hydropolitical approach may be useful to fully comprehend the underlying causes of the phenomena emerged in this work^67,68^, also in relation to the analysis of the consequences of the current COVID-19 pandemic on food trade and on the related virtual water displacement^69^.

Findings may be useful in the domain of policy, since they highlight the necessity to address water scarcity at a multidimensional level. Moreover, despite water scarcity is intrinsically a local phenomenon, the magnitude of the interconnections among countries with respect to scarce VW flows calls for an approach to the problem at a global scale. The present work is essential to quantify to what extent the addition of the economic water scarcity dimension to the physical water scarcity level produces results that change the perception of the virtual water trade patterns and dynamics. Hydropolitical lens would complement this approach in order to fully comprehend the underlying geopolitical and economic causes of the data-driven results that we highlighted. The interruption of environmentally or economically unfair VW exchanges may not be neither realistic, nor desirable, given the crucial role that food trade plays for food availability and variety in every country^70^. Therefore, a policy approach for addressing unsustainable and unfair VW trajectories may be that of intensifying international cooperation from the major recipient countries of scarce water to the origin countries with the largest gap in terms of economic water scarcity, that is to say in terms of water availability and access. Such cooperation initiatives could be even more appropriate in the water management and water infrastructural domain with the objective to reduce the divide to the maximum possible extent. Finally, findings may be useful for raising awareness among consumers, also through product labelling on the degree of fairness and sustainability of water use.

Future research would be highly appropriate in the study of the disaggregation of VW fluxes by single crops, in order to identify along which specific products physically- and economically-scarce virtual water is flowing.

## Data and methods

### Crop selection and the virtual water trade matrix

We select all the primary crops traded internationally—with the exclusion of live animals—for a total of 123 crops (see Supplementary Table S5). We utilize the virtual water (VW) trade matrix for the year 2016, which is the last year for which data are available in the CWASI database developed by Tamea et al.^46^. We sum the virtual water associated to the trade of each selected product, obtaining the total volumes of virtual water associated to primary crops exchanged by every country pair. We exclude countries involved in the trade having less than 1 million inhabitants (Supplementary Table S4). Therefore, in our matrix we include 22,200 VW fluxes that overall include 1.27E + 12 cubic meters of virtual water.

### The calculation of the crop unit water footprint

The volumes of the virtual water trade are calculated by multiplying the unit water footprint of the single crop in a specific country and year by the tonnes of the same crop exported and/or imported by the country in the same year. For each crop, the unit water footprint (*uWF*) is the ratio between the crop evapotranspiration (*ET*, in mm; e.g. the water consumed by the plant during the growing season), and the crop yield, *Y* (in ton/ha), i.e.

6 $$ uWF = 10 \cdot ET/Y $$

where the factor 10 converts the units of *uWF* into m^3^/ha. The lower is the *uWF* value, the more efficient is the use of water resources in crop production. The water consumed by the crop and lost through evapotranspiration could be originated from rainfall (green water), or from irrigation (blue water), which in turn may be originated from surface or groundwater. We consider the sum of blue and green WF. The WF is crop and country specific, since it depends on the crop features, on the climatic conditions, and on the cultivation techniques. The CWASI database includes data on the *uWF* for each crop in each country over the period 1961–2016^46^.

### Other data

The Integrated Water Resources Management (IWRM) indicator, that we utilize for the economic water scarcity component of our composite water scarcity index, is part of the Sustainable Development Goal 6 dedicated to water resources. It is available for 2017, while indexes on water management based on slightly different sets of questions are available for some previous year as well^57^. The second round of data collection on SDG indicator 6.5.1 took place during 2020 and data analysis are currently underway by UNEP.

Considerations on the strengths and the potential sources of uncertainty of the IWRM indicator are extensively discussed in^53,57–60,71^. Its strength lies in the fact that it declares to provide a framework to assess whether, in a given country, water resources are developed, managed and used in an equitable, efficient, and sustainable mode, reflecting diverse aspects of water management and water governance. It is based on a set of questions posed to country representatives on four pillars of water management: enabling environment, institutions and participation, management instruments and financing. It covers therefore different domains of water governance and management, distinguishing itself from other indicators where water governance is related to one single dimension only, such as for example the legislative framework^43,54,55^, transboundary issues^55^, or the infrastructures^43,54,56,72^. Furthermore, the methodology for the indicator’s construction allows coherence among the weights assigned to the different sub-dimensions. This last issue was detected as a problem some other water poverty indicators^44^. Finally, the IWRM indicator brings new information with respect the most common measures of socio-economic development of a country, such as Gross Domestic Product per capita and the Human Development Index, and with respect to its water availability^53,57^. Nevertheless, the indicator contains some critical aspects^53^. First, it is compiled at the national scale and it gives neither information on internal variations of the pillars across provinces of the country, nor on transboundary water basins. However, it must be acknowledged that the most important decisions on water governance, such as the level of decentralization, are taken at the central government level, and this makes the indicator appropriate. Second, the construction methodology of the index translates qualitative information form the survey into quantitative scores, which produces uncertainty in measurement accuracy. Third, global comparison may be challenging, given the potentially different interpretations that different countries attach to the threshold levels of the different pillars. Finally, an accurate comparison overtime is equally challenging, since for a given country a progress could be given by a significant advancement with respect to its previous level of water governance, even if such level is still relatively low when compared to other countries. This calls for a general reflection on whether it is appropriate to set the same global targets for all nations, given the diversity of their initial situations.

Regarding a similar discussion on the index on pressure on hydrological resources, that we use as indicator of physical water scarcity, Lenzen et al.^27^ underline that this indicator, provided by AQUASTAT, is of immediate understanding. It overcomes the limit posed by other indicators that place water availability in relation to population in a country, and that assume that a fixed and universal average water demand threshold should be met in order to avoid water shortage (see also^52^). On the contrary, the selected indicator on water pressure is only sensitive to the national conditions, and it is therefore useful to identify countries with relatively high extraction in relation to available hydrological resources, quantifying the respective water stress. The indicator is useful to express the notion that water utilization in a country with higher water stress is more valued than water utilization in a country with lower water stress. Damkjaer and Taylor^52^ report that scholars agreed on the following stress thresholds based on numerous sensitivity analysis. A country is considered ‘water stressed’ if annual withdrawals are between 20 and 40% of annual freshwater supply and ‘severely stressed’ if this figure exceeds 40%. Nevertheless, the indicator presents some shortcomings that may become a source of uncertainty^27,52^. First, it measures only groundwater withdrawals without covering rainfall, which counts indeed for a large part of total water use^1^. However, Lenzen et al.^27^ highlight that in reality farmers use extracted groundwater only after rainfall supply has run out, therefore focusing on groundwater stress is an appropriate way to measure water scarcity in a country. Second, it masks inter-annual variability in freshwater resources. Third, water withdrawal data do not take into account how much of it is used for consumption and how much could remain available for return flows. Fourth, the indicator does not inform on a society’s adaptive capacity to cope with water stress.

Summing up, given the limits and the complexity of the measurements of the parameters on physical and economic water scarcity, it is clear that we inherit such uncertainty and intrinsic difficulty of evaluation in the composite water scarcity index that we propose in the present paper. Therefore, further research on this issue is surely needed.

For the classification of countries in income categories we refer to the 2016 taxonomy developed by The World Bank^65^, on the basis of the Gross National Income per capita (GNI per capita in US$—Atlas methodology). From the same source we also retrieve the figures on population for the same year. In the Supplementary Material we include the complete list of countries included in the study.

In Fig. 7 and in Supplementary Figure S3 we investigate more in depth the link between trade of scarce virtual water and socio-economic vulnerability of a country and we select the 40 countries with the highest prevalence of undernourishment, and with a composite water scarcity index higher than 0.5. We measure food security for the so-called fragile countries through the indicator of prevalence of undernourishment in 2016, provided by the database of World Development Indicators of The World Bank^64^, Although the prevalence of undernourishment does not perfectly overlap with the concept of food security, since it captures the access to a sufficient energy intake but it disregards other relevant dimensions, it was chosen by the United Nations as the main indicator of the food security of total population to measure the progress toward the “Zero Hunger” Goal (Sustainable Development Goal number 2). It is defined as “an estimate of the proportion of the population whose habitual food consumption is insufficient to provide the dietary energy levels that are required to maintain a normal active and healthy life”^73^. In the scientific literature on food security this indicator is often adopted^74–77^, since it presents the advantage of not being affected by the distributional issues that are hidden beyond average values, as it happens for example in the case of calorie availability per capita^78^.

### Methods

We calculate the status of net VW export or net VW import of each country as

7 $$ N_{i} = E_{i} - I_{i} $$

where *E*_*i*_ and *I*_*i*_ represent respectively the VW export and the VW import of country *i*.

Analogously we perform the same calculation utilizing the CWSI-weighted VW trade values (Eqs. 4 and 5), denoted with *W* in the following equation

8 $$ WN_{i} = WE_{i} - WI_{i} $$

In order to allow the comparison between the volumetric and the weighted VW status, we normalize the results by dividing them for the sum (over all countries) of their absolute values, as

9 $$ SN_{i} = \frac{{N_{i} }}{{\mathop \sum \nolimits_{j} \left| {N_{j} } \right|}} $$

for volumetric values, and as

10 $$ SWN_{i} = \frac{{WN_{i} }}{{\mathop \sum \nolimits_{j} \left| {WN_{j} } \right|}} $$

for the scarcity-weighted values.

For the correct comparison of figures on net VW trade per capita we compute normalized values in the following ways. We first compute the volumetric VW export (*e*_*i*_) and import (*i*_*i*_) per capita for each country, by dividing its total export and import of VW associated to the 123 primary crops by its population (lower case letters indicate per capita figures). We then compute the same figure for the scarcity-weighted export and import per capita of each country (respectively, *we*_*i*_ and *wi*_*i*_). Subsequently, the net VW trade status of each country is computed as

11 $$ n_{i} = \frac{{E_{i} - I_{i} }}{{P_{i} }} $$

for the volumetric case, and as

12 $$ wn_{i} = \frac{{WE_{i} - WI_{i} }}{{P_{i} }} $$

for the scarcity-weighted figures.

Finally, we calculate the share of each country’s net VW trade with respect to the world total as

13 $$ sn_{i} = \frac{{n_{i} }}{{\mathop \sum \nolimits_{j} \left| {n_{j} } \right|}} $$

14 $$ swn_{i} = \frac{{wn_{i} }}{{\mathop \sum \nolimits_{j} \left| {wn_{j} } \right|}} $$

Moreover, we aim at comparing the VW volumes weighted for the CWSI index with the volumes weighted separately for physical scarcity and economic scarcity. Therefore, we repeat the whole procedure explained above by substituting the CWSI with, respectively, the physical water scarcity index (PWS) and the economic water scarcity index (EWS, expressed as 1-IWRM) in Eqs. (4) and (5).

## Supplementary Information

Supplementary Information.

## Data Availability

All data utilized in the study are publicly available.
