# Supplementary material for: Trade of economically and physically scarce virtual water in the global food network
Source: Sci Rep. 2021 Nov 23;11:22806. doi: 10.1038/s41598-021-01514-w (PMC8611042; doi:10.1038/s41598-021-01514-w)
Supplement: Supplementary file 1 — Supplementary Information. [file 41598_2021_1514_MOESM1_ESM.pdf]

TRADE OF ECONOMICALLY AND PHYSICALLY SCARCE VIRTUAL WATER IN THE GLOBAL FOOD NETWORK

Elena Vallino, Luca Ridolfi, Francesco Laio

Department of Environment, Land and Infrastructure Engineering
Politecnico di Torino, Italy

SUPPLEMENTARY MATERIAL

Figure S1. Scatterplot between the gap exporter-importer for each bilateral VW flux regarding the composite water scarcity index (CWSI, 2017) and income (GNI per capita in USD, Atlas method, 2016). Each dot represents one bilateral VW flux that includes all primary crops, for a total of 22,200 fluxes. Dot colors refer to the bar on the right. A lighter color corresponds to a larger flux (in log m3), and vice versa. We observe a concentration of large fluxes in quarter IV, in which water-related and economic unfairness is at the highest levels. (squaredR = 0.19). Sources: The World Bank (2020b), authors' elaboration.

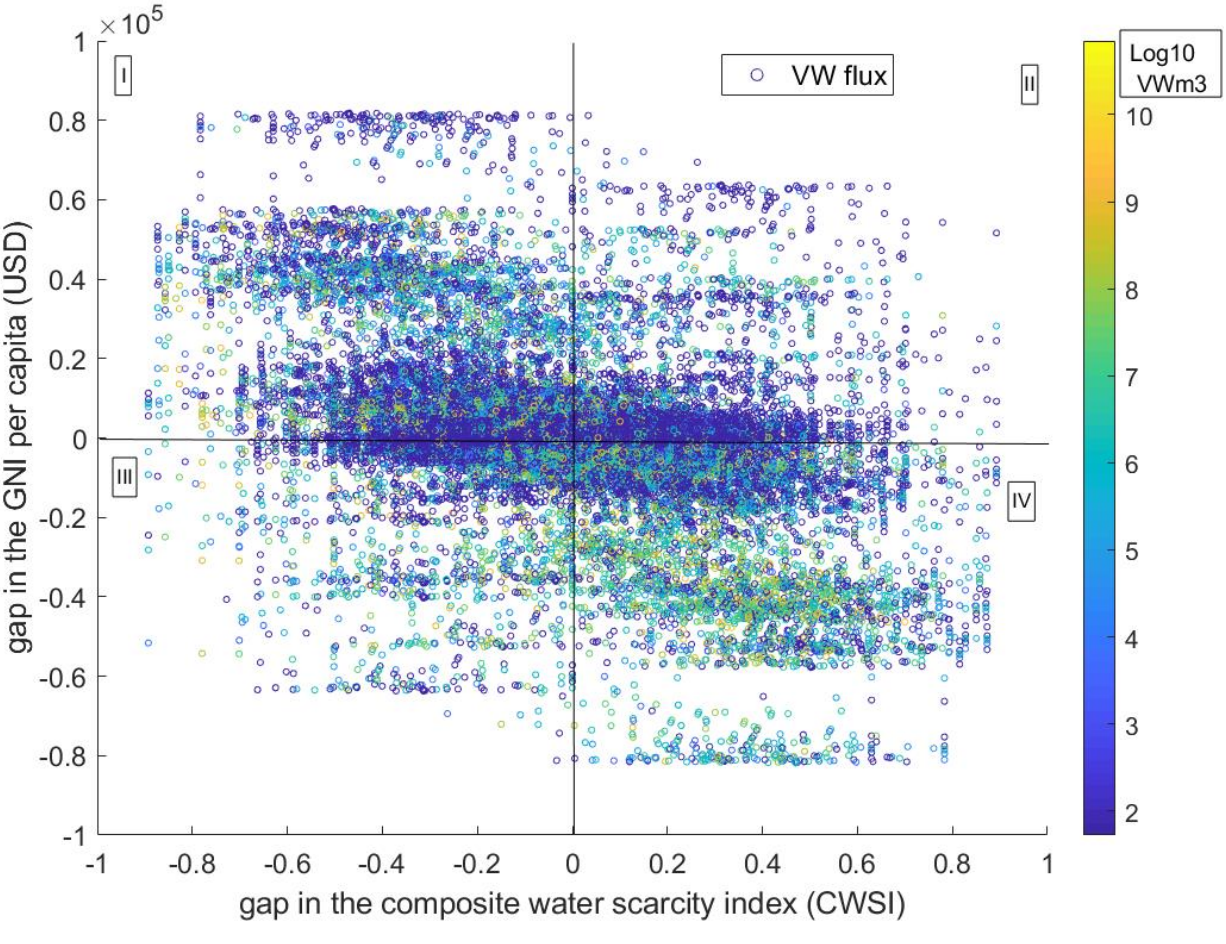

Table S1. Largest 20 volumetric and CWSI-weighted virtual water fluxes (2016)

Volumetric VW fluxes				CWSI-weighted VW fluxes			
Origin	Destination	m3	% on world total	Origin	Destination	weighted m3	% on world total
Brazil	China	7.04E+10	5.54	Brazil	China	3.5E+10	5.80
USA	China	5.01E+10	3.94	Indonesia	India	1.39E+10	2.31
Indonesia	India	2.43E+10	1.91	USA	China	1.33E+10	2.20
USA	Mexico	1.87E+10	1.47	Argentina	China	8.8E+09	1.46
Indonesia	China	1.39E+10	1.10	Indonesia	China	7.97E+09	1.32
Argentina	China	1.39E+10	1.09	Malaysia	India	5.97E+09	0.99
USA	Japan	1.36E+10	1.07	Indonesia	Pakistan	5.09E+09	0.84
Canada	China	1.09E+10	0.86	USA	Mexico	4.96E+09	0.82
Netherlands	Germany	1.04E+10	0.82	Argentina	Brazil	4.93E+09	0.82
Malaysia	India	1.03E+10	0.81	Malaysia	China	4.17E+09	0.69
Australia	China	9.85E+09	0.77	Thailand	China	4.15E+09	0.69
Russia	Egypt	9.18E+09	0.72	Thailand	Malaysia	3.92E+09	0.65
Indonesia	Pakistan	8.91E+09	0.70	Indonesia	USA	3.87E+09	0.64
Argentina	Brazil	7.78E+09	0.61	Ivory Coast	Netherlands	3.84E+09	0.64
Brazil	Iran	7.61E+09	0.60	Brazil	Iran	3.79E+09	0.63
Thailand	China	7.56E+09	0.59	Ivory Coast	Algeria	3.71E+09	0.61
Malaysia	China	7.23E+09	0.57	Kazakhstan	Uzbekistan	3.65E+09	0.61
Thailand	Malaysia	7.15E+09	0.56	USA	Japan	3.6E+09	0.60
Australia	Indonesia	6.9E+09	0.54	Mexico	USA	3.42E+09	0.57
Indonesia	USA	6.77E+09	0.53	Ivory Coast	India	3.4E+09	0.56

Table S2. Top 10 countries for net VW export and import, in volumetric values (Equation 7 and 9 in Data and Methods) and weighted for the composite water scarcity index, for the physical scarcity index and for the economic scarcity index (Equations 4 and 5), 2016. The upper layer is referred to absolute figures (percentage on the world total, Eqs 8 and 10). The bottom layer is referred to per capita figures (percentage on the world total, Eqs. 11, 12 and 13).

		No weight		Composite scarcity weight		Physical scarcity weight		Economic scarcity weight	
Total (world share)	Net exp	Brazil	9.27	Indonesia	9.70	USA	13.96	Brazil	11.00
		USA	8.24	Brazil	9.46	India	8.22	Indonesia	10.41
		Indonesia	7.40	Argentina	5.71	Indonesia	7.91	Argentina	6.46
		Argentina	4.27	Ivory Coast	4.03	Pakistan	6.47	Ivory Coast	4.75
		Australia	3.73	Malaysia	2.78	Thailand	3.35	Malaysia	3.54
		Canada	3.72	Thailand	2.66	Turkmenistan	3.18	Ukraine	2.74
		Russian F.	2.77	Ukraine	2.41	Kazakhstan	2.02	Thailand	2.68
		Ivory Coast	2.73	USA	2.04	Argentina	1.80	Paraguay	2.33
		Thailand	2.15	Paraguay	2.01	Un. Arab Em.	1.63	Kazakhstan	2.00
		Malaysia	2.15	Kazakhstan	1.84	Bulgaria	1.34	Canada	1.69
	Net imp	China	-16.01	China	-15.08	China	-11.05	China	-15.65
		Japan	-3.38	Netherlands	-4.33	Turkey	-3.37	Netherlands	-4.88
		Germany	-3.08	Germany	-3.13	Japan	-2.51	Germany	-3.35
		Netherlands	-2.62	Japan	-2.74	Germany	-2.01	Japan	-2.71
		Italy	-2.18	Turkey	-2.26	Iran	-2.00	Spain	-2.33
		Turkey	-2.07	Italy	-2.09	Malaysia	-1.99	Italy	-2.16
		Spain	-1.94	Spain	-2.01	Bangladesh	-1.80	Turkey	-2.09
		Egypt	-1.92	Korea (Rep)	-1.68	Saudi Arabia	-1.69	India	-1.87
		Korea, Rep	-1.82	Iran	-1.62	Italy	-1.65	Korea (Rep)	-1.72
		Viet Nam	-1.68	Egypt	-1.46	Netherlands	-1.47	Egypt	-1.65
Per capita (world share)	Net exp	Uruguay	5.32	Paraguay	6.80	Turkmenistan	13.65	Paraguay	7.26
		Paraguay	5.09	Ivory Coast	3.88	Bulgaria	4.58	Ivory Coast	4.22
		Australia	3.82	Uruguay	3.87	Un. Arab Em.	4.22	Uruguay	4.00
		Ivory Coast	2.84	Argentina	3.01	Kazakhstan	2.76	Argentina	3.13
		Canada	2.55	Moldova	2.89	Singapore	2.33	Moldova	3.01
		Lithuania	2.44	Turkmenistan	2.84	Estonia	2.00	Malaysia	2.44
		Argentina	2.42	Kazakhstan	2.37	Moldova	1.25	Kazakhstan	2.38
		Moldova	2.08	Lithuania	2.14	Thailand	1.18	Lithuania	2.36
		Malaysia	1.73	Malaysia	2.08	USA	1.05	Honduras	1.85
		Bulgaria	1.68	Bulgaria	1.89	Uruguay	1.04	Liberia	1.75
	Net imp	Netherlands	-3.81	Netherlands	-5.84	Oman	-3.54	Netherlands	-6.06
		Belgium	-3.13	Belgium	-2.70	Qatar	-2.80	Singapore	-3.49
		Singapore	-2.72	Singapore	-2.51	Benin	-2.38	Belgium	-2.84
		Un. Arab Em.	-1.66	Oman	-1.45	Netherlands	-2.10	Un. Arab Em.	-1.73
		Israel	-1.34	Benin	-1.11	Botswana	-1.69	Spain	-1.06
		Oman	-1.30	Mauritius	-1.10	Malaysia	-1.58	Mauritius	-0.99
		Saudi Arabia	-1.11	Qatar	-1.02	Mauritius	-1.47	Oman	-0.95
		Kuwait	-1.10	Saudi Arabia	-1.01	Kuwait	-1.47	Saudi Arabia	-0.91
		Mauritius	-1.05	Spain	-0.99	Saudi Arabia	-1.27	Benin	-0.89
		Spain	-1.03	Portugal	-0.97	Gambia	-1.12	Portugal	-0.87

Fig. S2. Largest 20 net CWSI-weighted VW importers per capita (negative values for swn_i) and largest 20 net CWSI-weighted VW exporters per capita (positive values for swn_i), expressed as world share, 2016. Net trade is calculated as export minus import.

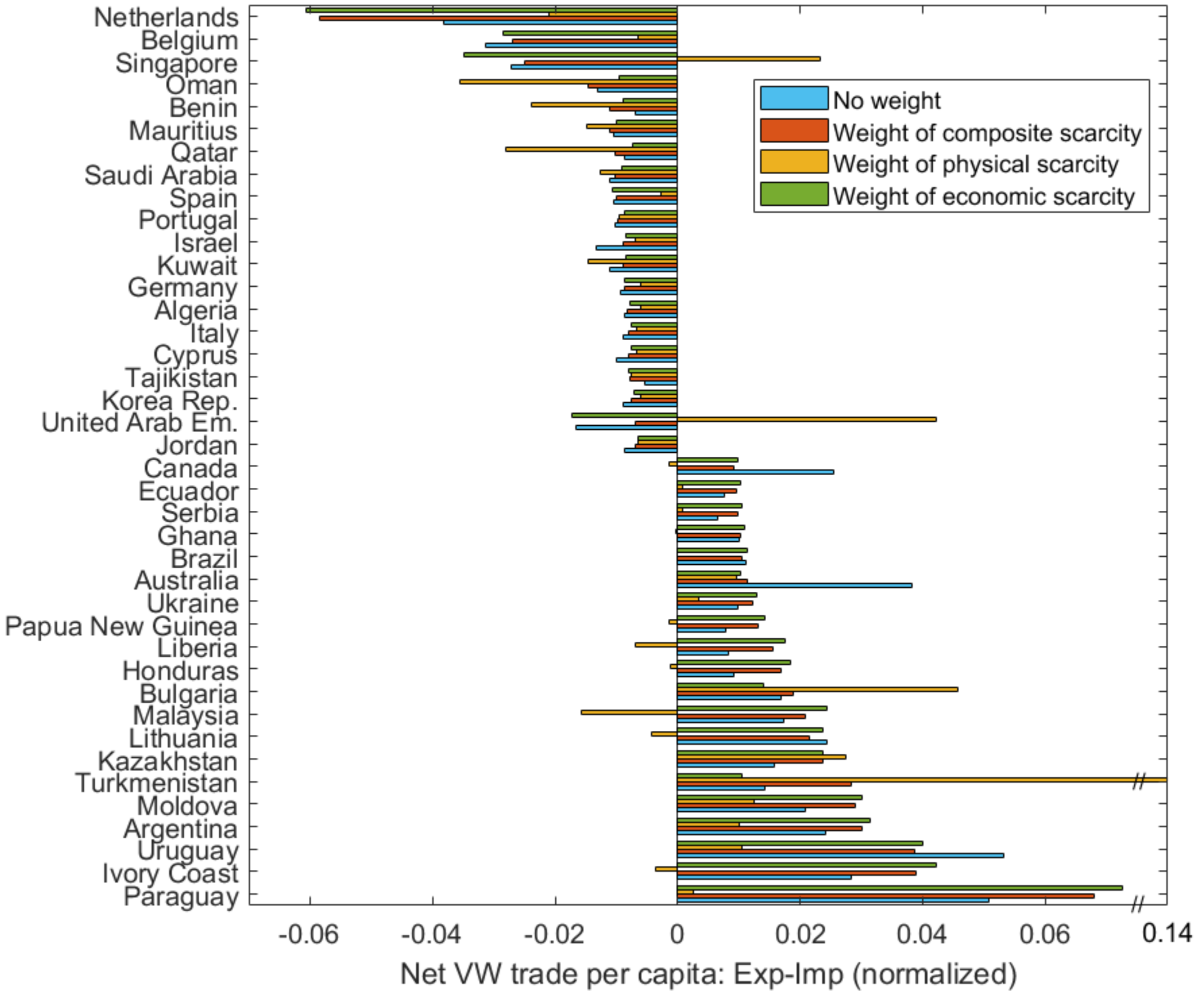

Table S3. **(A)** Gap between the volumetric VW and the economic-scarcity weighted VW for all net exporters per capita (share on the world total, 2016). Negative gap: the net export for economic-scarcity-weighted VW is higher than for the volumetric VW, and *vice versa*. Underlined countries become net importers if the economic scarcity weight is applied. **(B)** Gap between the volumetric VW and the economic-scarcity weighted VW for the all importers per capita (share on the world total, 2016). Positive gap: the net import for economic-scarcity-weighted VW is higher than for the volumetric VW, and *vice versa*. Underlined countries become net exporters if the economic scarcity weight is applied.

(A) Net exporters per capita	Gap Volumetric VW / economic-scarcity-weighted VW	(B) Net importers per capita	Gap Volumetric VW / economic-scarcity-weighted VW
Paraguay	-0.0217	Netherlands	0.0225
Côte d'Ivoire	-0.0138	Singapore	0.0078
Moldova Rep	-0.0093	Tajikistan	0.0026
Liberia	-0.0092	Denmark	0.0025
Honduras	-0.0092	Greece	0.0022
Kazakhstan	-0.0081	Benin	0.0021
Malaysia	-0.0071	Uzbekistan	0.0012
Argentina	-0.0071	Switzerland	0.0008
Papua New Guinea	-0.0064	United Arab Em.	0.0007
Costa Rica	-0.0052	United Kingdom of Great Britain and Northern Ireland	0.0004
Guatemala	-0.0052	Sweden	0.0004
Serbia	-0.0041	South Africa	0.0003
Togo	-0.0032	Spain	0.0002
Ukraine	-0.0030	Syrian Arab Rep.	0.0002
Sri Lanka	-0.0027	Bosnia and Herz.	0.0002
Ecuador	-0.0026	India	0.0001
Poland	-0.0026	Angola	0.0000
Cameroon	-0.0023	Finland	0.0000
Timor-Leste	-0.0021	Eritrea	0.0000
Nicaragua	-0.0019	<u>Korea, DPR</u>	-0.0001
Colombia	-0.0015	<u>Congo, DR</u>	-0.0001
Indonesia	-0.0014	Mozambique	-0.0001
Ghana	-0.0010	Pakistan	-0.0002
Nigeria	-0.0008	Nepal	-0.0002
Ethiopia	-0.0006	Kenya	-0.0002
Thailand	-0.0005	Afghanistan	-0.0002
Madagascar	-0.0004	Zimbabwe	-0.0002
Malawi	-0.0004	Gambia	-0.0002
Chad	-0.0003	Belarus	-0.0003
Central African Rep	-0.0003	Eq. Guinea	-0.0003
Rwanda	-0.0003	Senegal	-0.0004
Cambodia	-0.0002	Somalia	-0.0004
Burundi	-0.0002	Iran	-0.0004
Zambia	-0.0002	China	-0.0005
Tanzania	-0.0002	Lao, PDR	-0.0005
Brazil	-0.0001	Iraq	-0.0005
Niger	-0.0001	Bangladesh	-0.0005
Bolivia	0.0000	Cuba	-0.0005
Mali	0.0001	Myanmar	-0.0005
Slovakia	0.0002	Sierra Leone	-0.0006
Uganda	0.0002	Mauritius	-0.0006

Latvia	0.0003	Albania	-0.0006
CONT. (A)		CONT (B)	
Lithuania	0.0008	Germany	-0.0007
Burkina Faso	0.0013	Congo	-0.0007
Czech Republic	0.0016	Slovenia	-0.0007
Bulgaria	0.0028	Philippines	-0.0007
Croatia	0.0028	Lesotho	-0.0007
Turkmenistan	0.0038	Venezuela	-0.0008
Hungary	0.0041	Algeria	-0.0008
Russian Federation	0.0043	Turkey	-0.0009
Romania	0.0064	Namibia	-0.0009
France	0.0064	North Macedonia	-0.0010
USA	0.0070	Tunisia	-0.0011
Estonia	0.0105	Haiti	-0.0012
Uruguay	0.0132	Mongolia	-0.0012
Canada	0.0156	Armenia	-0.0012
Australia	0.0279	Guinea	-0.0013
		Chile	-0.0013
		Gabon	-0.0013
		Mauritania	-0.0013
		Qatar	-0.0013
		Egypt	-0.0013
		Italy	-0.0014
		Botswana	-0.0015
		Norway	-0.0015
		Portugal	-0.0015
		Korea, Rep.	-0.0017
		Azerbaijan	-0.0017
		Yemen	-0.0017
		Viet Nam	-0.0019
		Saudi Arabia	-0.0019
		Austria	-0.0020
		Jamaica	-0.0020
		Morocco	-0.0021
		Japan	-0.0021
		Jordan	-0.0021
		Ireland	-0.0021
		New Zealand	-0.0022
		Panama	-0.0023
		Cyprus	-0.0024
		Mexico	-0.0024
		Trinidad and Tobago	-0.0024
		Dominican Rep.	-0.0024
		Georgia	-0.0024
		Libya	-0.0025
		Kuwait	-0.0026
		Peru	-0.0028
		El Salvador	-0.0028
		Belgium	-0.0028
		Lebanon	-0.0030
		Oman	-0.0035
		Israel	-0.0048

Figure S3. Net VW trade per capita status for 40 countries with the highest prevalence of undernourishment among the overall population, and with a composite water scarcity index higher than 0.5 (range: 0-1), 2016. Countries are ranked according to the share of CWSI-weighted VW trade per capita. Net trade is calculated as export minus import.

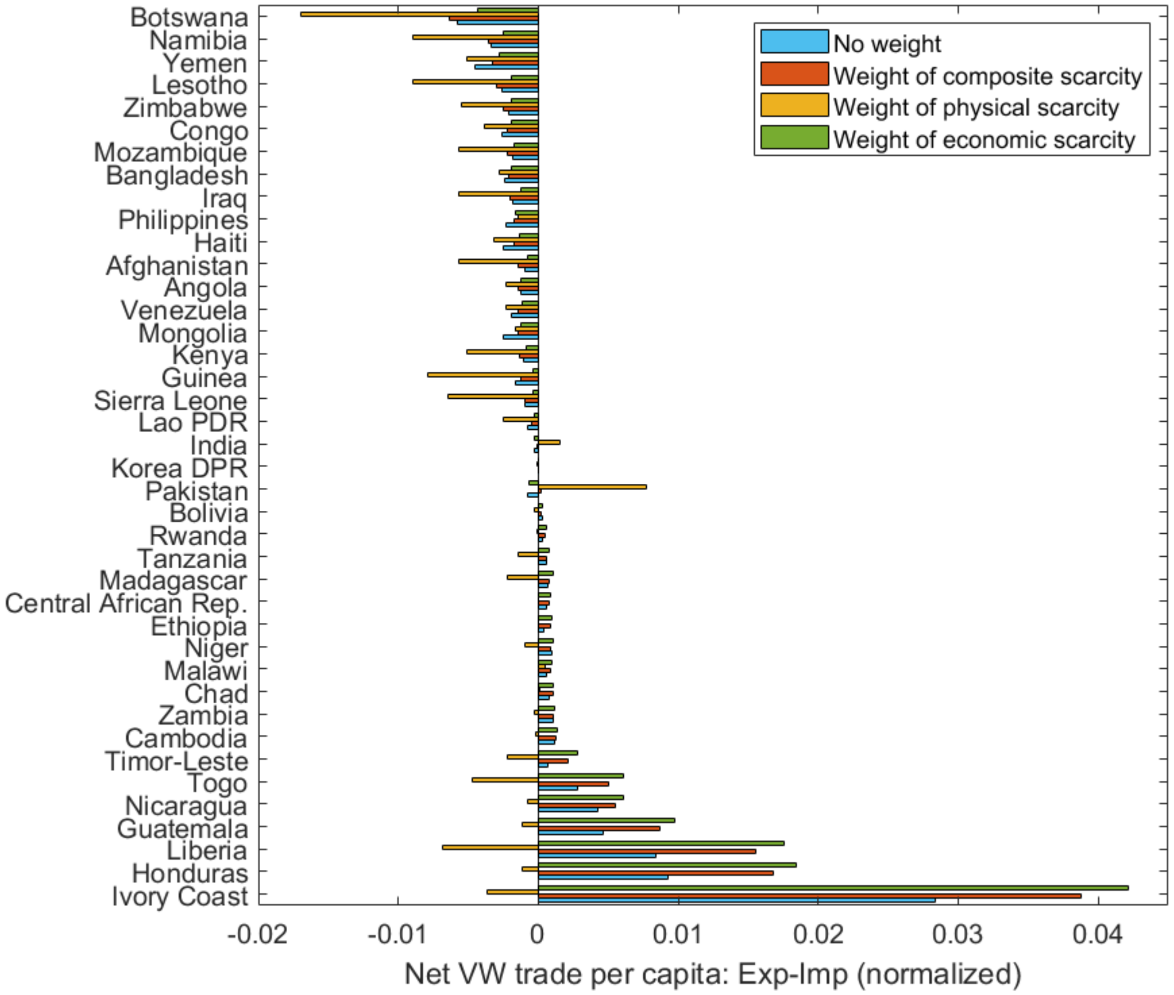

Table S4. Countries with indexes of composite water scarcity, physical water scarcity (2017), economic water scarcity (2017), GNI per capita (Atlas method, USD, 2016). The three indexes lie in the range 0-1, where 0 denotes absence of scarcity and 1 denotes maximum scarcity. Sources: Aquastat (2020), UN-Environment (2018), The World Bank (2020b), authors' elaboration.

	Composite scarcity index	Physical scarcity index	Economic scarcity index	GNIpc (USD; 2016)
Afghanistan	0.92	0.31	0.88	570
Albania	0.59	0.04	0.57	4320
Algeria	0.92	0.84	0.52	4370
Angola	0.63	0.00	0.63	3770
Argentina	0.63	0.04	0.62	12220
Armenia	0.77	0.37	0.64	3760
Australia	0.17	0.03	0.15	54140
Austria	0.13	0.04	0.09	46220
Azerbaijan	0.58	0.37	0.34	4760
Bangladesh	0.51	0.03	0.50	1370
Belarus	0.63	0.02	0.62	5620
Belgium	0.39	0.22	0.22	43440
Benin	0.38	0.00	0.37	1110
Bolivia	0.51	0.00	0.51	3040
Bosnia and Herzegovina	0.40	0.01	0.39	5130
Botswana	0.60	0.02	0.59	6570
Brazil	0.50	0.01	0.49	8920
Bulgaria	0.56	0.27	0.40	7430
Burkina Faso	0.41	0.06	0.37	660
Burundi	0.69	0.02	0.68	270
Cambodia	0.55	0.00	0.54	1140
Cameroon	0.66	0.00	0.66	1380
Canada	0.23	0.01	0.22	43870
Central African Republic	0.69	0.00	0.69	410
Chad	0.69	0.02	0.68	730
Chile	0.78	0.04	0.77	13370
China	0.41	0.21	0.26	8270
Colombia	0.50	0.00	0.50	6460
Congo	0.68	0.00	0.68	1820
Congo, Democratic Republic	0.69	0.00	0.69	470
Costa Rica	0.58	0.03	0.57	10770
Côte d'Ivoire	0.68	0.01	0.68	1840
Croatia	0.11	0.01	0.10	12380
Cuba	0.34	0.18	0.20	7480
Cyprus	0.34	0.28	0.09	24700
Czech Republic	0.31	0.12	0.21	17590
Denmark	0.18	0.12	0.07	58040
Dominican Republic	0.75	0.30	0.64	6860
Ecuador	0.59	0.02	0.58	5810
Egypt	1.00	1.00	0.60	3460
El Salvador	0.80	0.08	0.79	3510
Equatorial Guinea	0.76	0.00	0.76	8270

Eritrea	0.34	0.08	0.28	600
Estonia	0.31	0.14	0.20	18280
Ethiopia	0.71	0.09	0.69	670
Finland	0.30	0.06	0.25	45490
France	0.14	0.13	0.02	39070
Gabon	0.86	0.00	0.86	7080
Gambia	0.71	0.01	0.70	620
Georgia	0.66	0.03	0.65	4080
Germany	0.26	0.16	0.12	44290
Ghana	0.52	0.02	0.51	1820
Greece	0.30	0.16	0.17	18890
Guatemala	0.76	0.03	0.75	3870
Guinea	0.76	0.00	0.76	780
Haiti	0.74	0.10	0.71	780
Honduras	0.80	0.02	0.80	2120
Hungary	0.30	0.04	0.27	13050
India	0.64	0.34	0.46	1680
Indonesia	0.57	0.11	0.52	3400
Iran	0.81	0.68	0.41	5500
Iraq	0.86	0.43	0.75	5620
Ireland	0.21	0.01	0.20	52490
Israel	0.72	0.67	0.15	36360
Italy	0.55	0.18	0.45	31950
Jamaica	0.62	0.13	0.57	4600
Japan	0.24	0.19	0.06	37860
Jordan	0.98	0.96	0.37	3930
Kazakhstan	0.76	0.21	0.70	8770
Kenya	0.54	0.13	0.47	1350
Korea, Republic	0.60	0.42	0.32	29330
Kuwait	1.00	1.00	0.19	36880
Lao People's Democratic Rep.	0.64	0.01	0.64	2120
Latvia	0.36	0.01	0.36	14600
Lebanon	0.81	0.40	0.68	7230
Lesotho	0.68	0.01	0.67	1300
Liberia	0.85	0.00	0.85	620
Libya	1.00	1.00	0.53	4550
Lithuania	0.44	0.01	0.43	14870
Macedonia, The former YR	0.79	0.08	0.78	4980
Madagascar	0.65	0.04	0.64	470
Malawi	0.63	0.08	0.60	340
Malaysia	0.58	0.01	0.57	10150
Mali	0.49	0.04	0.47	780
Mauritania	0.60	0.12	0.55	1510
Mauritius	0.50	0.22	0.36	10640
Mexico	0.60	0.19	0.51	9400
Moldova, Republic	0.71	0.07	0.68	3180
Mongolia	0.58	0.01	0.57	3500
Morocco	0.59	0.36	0.36	2880
Mozambique	0.46	0.01	0.45	530

Myanmar	0.73	0.03	0.73	1280
Namibia	0.41	0.01	0.41	4700
Nepal	0.69	0.05	0.67	770
Netherlands	0.15	0.09	0.07	46900
New Zealand	0.44	0.03	0.42	39390
Nicaragua	0.58	0.01	0.58	2000
Niger	0.53	0.05	0.50	500
Nigeria	0.66	0.04	0.65	2470
Norway	0.37	0.01	0.37	81810
Oman	1.00	1.00	0.67	15810
Pakistan	0.91	0.81	0.50	1420
Panama	0.64	0.01	0.63	12780
Papua New Guinea	0.75	0.00	0.75	2700
Paraguay	0.68	0.01	0.68	5390
Peru	0.71	0.01	0.70	6110
Philippines	0.59	0.19	0.49	3450
Poland	0.67	0.17	0.61	12680
Portugal	0.35	0.12	0.26	19930
Qatar	1.00	1.00	0.18	64010
Romania	0.30	0.03	0.28	9650
Russian Federation	0.22	0.01	0.21	9730
Rwanda	0.66	0.01	0.65	750
Saudi Arabia	1.00	1.00	0.43	21610
Senegal	0.50	0.06	0.47	1270
Serbia	0.71	0.03	0.70	5690
Sierra Leone	0.81	0.00	0.81	490
Singapore	0.84	0.83	0.02	53020
Slovakia	0.35	0.01	0.34	16790
Slovenia	0.44	0.03	0.42	21750
South Africa	0.59	0.38	0.35	5470
Spain	0.41	0.28	0.18	27530
Sri Lanka	0.81	0.25	0.75	3810
Sweden	0.13	0.01	0.12	54370
Switzerland	0.22	0.04	0.19	82110
Syrian Arab Republic	0.89	0.84	0.30	1820
Tajikistan	0.81	0.51	0.61	1100
Tanzania	0.52	0.05	0.50	970
Thailand	0.55	0.13	0.48	5700
Timor-Leste	0.88	0.14	0.86	1750
Togo	0.68	0.01	0.68	610
Trinidad and Tobago	0.77	0.09	0.75	16720
Tunisia	1.00	1.00	0.46	3750
Turkey	0.50	0.28	0.31	11180
Turkmenistan	1.00	1.00	0.35	6820
Uganda	0.42	0.01	0.41	790
Ukraine	0.63	0.05	0.61	2370
United Arab Emirates	1.00	1.00	0.25	40570
United Kingdom	0.28	0.06	0.23	42980
USA	0.26	0.14	0.14	57180

Uruguay	0.39	0.02	0.38	14980
Uzbekistan	1.00	1.00	0.55	2660
Venezuela	0.54	0.02	0.53	13080
Viet Nam	0.66	0.09	0.62	2080
Yemen	1.00	1.00	0.61	1070
Sudan	0.89	0.71	0.60	1570
South Sudan	0.62	0.01	0.62	1090
Zambia	0.55	0.02	0.54	1370
Zimbabwe	0.49	0.17	0.39	1290

Table S5. Primary crops included in the study. Source: Tamea et al. (2021).

FAO code	Product
15	Wheat
27	Rice, paddy
44	Barley
56	Maize
71	Rye
75	Oats
79	Millet
83	Sorghum
89	Buckwheat
94	Fonio
97	Triticale
101	Canary seed
103	Mixed grain
116	Potatoes
122	Sweet potatoes
125	Cassava
149	Roots and Tubers, nes
157	Sugar beet
161	Sugar crops, nes
176	Beans, dry
181	Broad beans, horse beans, dry
187	Peas, dry
191	Chick peas
201	Lentils
203	Bambara beans
205	Vetches
217	Cashew nuts, with shell
220	Chestnuts
222	Walnuts, with shell
223	Pistachios
224	Kolanuts
234	Nuts, nes
236	Soybeans
249	Coconuts
257	Palm oil
258	Oil, palm kernel
260	Olives
267	Sunflower seed
270	Rapeseed
289	Sesame seed
292	Mustard seed
296	Poppy seed
311	Kapokseed in shell
329	Cottonseed
333	Linseed
339	Oilseeds, Nes

358	Cabbages and other brassicas
366	Artichokes
367	Asparagus
372	Lettuce and chicory
373	Spinach
388	Tomatoes
393	Cauliflowers and broccoli
394	Pumpkins, squash and gourds
397	Cucumbers and gherkins
399	Eggplant-baseds (aubergines)
401	Chillies and peppers, green
402	Onions (inc. shallots), green
403	Onions, dry
406	Garlic
407	Leeks, other alliaceous vegetables
414	Beans, green
417	Peas, green
426	Carrots and turnips
446	Maize, green
449	Mushrooms and truffles
463	Vegetables fresh nes
486	Bananas
489	Plantains
490	Oranges
495	Tangerines, mandarins, clem.
497	Lemons and limes
507	Grapefruit (inc. pomelos)
515	Apples
521	Pears
523	Quinces
526	Apricots
530	Sour cherries
531	Cherries
534	Peaches and nectarines
536	Plums and sloes
544	Strawberries
549	Gooseberries
550	Currants
552	Blueberries
554	Cranberries
560	Grapes
567	Watermelons
568	Other melons (inc.cantaloupes)
569	Figs
571	Mangoes, mangosteens, guavas
572	Avocados
574	Pineapples
577	Dates
587	Persimmons

591	Cashew apple
592	Kiwi fruit
600	Papayas
603	Fruit, tropical fresh nes
619	Fruit Fresh Nes
656	Coffee, green
661	Cocoa beans
667	Tea
671	Maté
677	Hops
687	Pepper (Piper spp.)
689	Chillies and peppers, dry
692	Vanilla
693	Cinnamon (canella)
698	Cloves
702	Nutmeg, mace and cardamoms
711	Anise, badian, fennel, corian.
720	Ginger
723	Spices, nes
748	Peppermint
767	Cotton lint
770	Cotton linters
773	Flax fibre and tow
778	Kapok fibre
780	Jute
809	Manila Fibre (Abaca)
826	Tobacco, unmanufactured
836	Natural rubber

References

AQUASTAT (2020) Core Database, Food and Agriculture Organization of the United Nations.

Tamea, S., Tuninetti, M., Soligno, I., and Laio, F. (2021). Virtual water trade and water footprint of agricultural goods: the 1961–2016 CWASI database, *Earth System Science Data,* 13, 1–27.

The World Bank, 2020b, World Development Indicators, DataBank, https://databank.worldbank.org/source/world-development-indicators

UN Environment (2018) *Progress on integrated water resources management. Global baseline for SDG 6. Indicator 6.5.1: degree of IWRM implementation*. UNEP.
